# Psychological Hibernation in Antarctica

**DOI:** 10.3389/fpsyg.2018.02235

**Published:** 2018-11-20

**Authors:** Gro Mjeldheim Sandal, Fons J. R. van deVijver, Nathan Smith

**Affiliations:** ^1^Department of Psychosocial Science, University of Bergen, Bergen, Norway; ^2^Department of Culture Studies, Tilburg University, Tilburg, Netherlands; ^3^Department of Psychology, North-West University, Potchefstroom, South Africa; ^4^Department of Politics, University of Manchester, Manchester, United Kingdom

**Keywords:** Antarctica, coping strategies, affect, psychological resilience, winter-over syndrome

## Abstract

Human activity in Antarctica has increased sharply in recent years. In particular during the winter months, people are exposed to long periods of isolation and confinement and an extreme physical environment that poses risks to health, well-being and performance. The present study aimed to gain a better understanding of processes contributing to psychological resilience in this context. Specifically, the study examined how the use of coping strategies changed over time, and the extent to which changes coincided with alterations in mood and sleep. Two crews (*N* = 27) spending approximately 10 months at the Concordia station completed the Utrecht Coping List, the Positive and Negative Affect Schedule (PANAS), and a structured sleep diary at regular intervals (x 9). The results showed that several variables reached a minimum value during the midwinter period, which corresponded to the third quarter of the expedition. The effect was particularly noticeable for coping strategies (i.e., active problem solving, palliative reactions, avoidance, and comforting cognitions). The pattern of results could indicate that participants during Antarctic over-wintering enter a state of psychological hibernation as a stress coping mechanism.

## Introduction

Antarctica is the darkest, coldest and most hostile region for humans on Earth. Antarctica has no permanent residents, but many of the nations that are signatory to the Antarctic Treaty send personnel to conduct seasonal (summer) and all year round research on the continent and the Southern Ocean environments. Securing the human side of such expeditions is a matter of concern as many participants are exposed to challenging living and working conditions that pose risks to their safety, performance and psychological health. During the winter months, depending on the location, people may be totally physically isolated from the outside world, with darkness and weather conditions putting severe constraints on the possibility to travel. There are periods when supplies cannot be transported to the stations and evacuation in case of emergencies may not be possible. Although telecommunication between station and outside world has greatly improved in recent years, these systems may temporarily break down and outside experts may not be available for consultation at all times if help is needed. Key stressors for participants include lack of social variation, monotony of the physical environment, confinement and limited privacy, and emotional and physical deprivations. While there are large individual variations in psychological resilience, foregoing research has long since documented that personnel overwintering on Antarctic stations are vulnerable to mental and somatic health problems (Palinkas and Suedfeld, 2008; Arendt, 2012; Chen et al., 2016). Indeed, reductions in mood, adaptation problems, and sleep difficulties account for 60% of all diagnoses in Antarctica (Palinkas et al., 1995; Lugg, 2005). As these reactions seem most prevalent in the cold and dark season, this cluster of symptoms has been referred to as the ‘winter-over syndrome’ (Palmia, 1963; Palinkas et al., 1995).

Over the last decades, many research efforts have been dedicated to explain the nature and causes of seasonal variations in psychological resilience among personnel over-wintering in Antarctica. The concept of resilience refers to the human capacity to cope successfully (and even grow) under significant adverse conditions (Fletcher and Sarkar, 2013). Three different but potentially compatible mechanisms have been proposed to explain the winter-over syndrome and subsequent impacts upon a person’s capacity to maintain resilience in the face of stress. Firstly, the amount of daylight varies extremely across seasons on these latitudes. Antarctica has just two seasons: summer and winter. During summer at the poles, there is 6 months of constant sunlight and in winter there is 6 months of total darkness. These variations can significantly influence parameters such as circadian rhythms, sleep, mood, and executive functions (Beute and de Kort, 2014; LeGates et al., 2014; Collet et al., 2015; Steinach et al., 2016; Pattyn et al., 2018). Indeed, this explanation is supported by research that suggests exposure to blue-enriched (short-wavelength) light as a countermeasure may prevent circadian disorders among Antarctic over-winter personnel (Najjar et al., 2014). Secondly, studies have shown cold-related changes in thyroid function in Antarctica, referred to as the polar triiodothyronine (T3) syndrome, characterized by an increase in serum TSH, a decrease in free T4, and an increase in T3 production and clearance. These changes have typically been associated with decreased mood and cognitive impairment (Do et al., 2004; Chen et al., 2016). Thirdly, research across different confined and isolated contexts have suggested that psychological resilience is linked to the relative passage of time, and that a decrease tends to occur around the third quarter of the stay independent of the actual duration, as the participants realize that the mission is only half complete (Bechtel and Berning, 1991). For winter-over crews the third quarter coincides with midwinter, which may confound any difficulties experienced.

The third-quarter perspective suggests that psychological resilience to some extent is associated with expectancies about the duration of the stay. This idea is in line with contemporary stress models which emphasize that appraisal is critical to how individuals cope with challenging situations, in turn predicting physiological activation (e.g., elevated levels of adrenaline or cortisol, increased blood pressure and faster heartbeats), psychological responses (e.g., feeling of distress, anxiety, sleeping problems), and behavior (e.g., expression of tension toward crewmates). While data from several studies conducted among groups operating in polar environments have indicated declines in mood and moral around the third quarter (Stuster et al., 2000; Kjærgaard et al., 2013), empirical support remains equivocal. An alternative perspective has been that psychological resilience gradually declines as individual resources are depleted (Palinkas and Houseal, 2000) and thus, that there is a linear relationship between psychological resilience, experience of stress, and time (Nicholas et al., 2015). In contrast to prior explanations, other researchers have pointed out that many participants also experience positive (salutogenic) effects from successful adaptation to polar environments and report feelings of personal growth and improvements in health (Palinkas and Suedfeld, 2008).

Despite considerable research into how the Antarctic environment impacts on psychological resilience, the process by which people try to manage the demanding living conditions is not well understood. According to Fletcher and Sarkar (2013) psychological resilience influences the stress process at multiple stages, the individual’s appraisal of a potential stressor, his or her meta-cognitions in reaction to felt emotions, and the choice of coping strategies. Appropriate coping strategies serve to promote optimal levels of activation either by eliminating or changing the sources of stress (“problem focused coping”) or by decreasing the emotional reactivity (“emotion oriented coping”) (Lazarus and Folkman, 1984). A number of empirical studies have linked coping responses to stable dispositional characteristics, and researchers have showed that habitual strategies are associated with adaptation in confined and isolated environments (Sandal et al., 1999; Grant et al., 2007). In the present study, we argue that a contextual approach to coping behavior will provide a more refined understanding of the adaptation process among Antarctic over-winterers. It is important to note that expeditioners may respond differently in this context from how they normally approach challenging situations (Smith et al., 2017). Firstly, lengthy isolation, confinement, and demanding physical environments place limitations on certain coping strategies, and amplify the importance on others (Palinkas and Browner, 1995; Sandal et al., 1999). Secondly, expeditioners may adapt their coping response to the challenges associated with different parts of the sojourn. Researchers have noted that the cumulative effects of chronic stressors may lead to a depletion of resources, which in turn may result in a shift from active, problem-based coping to more passive, emotion-based strategies (Couch and Coles, 2011). According to transactional stress theory (Lazarus and Folkman, 1984), as a challenging situation develops, there is a continuing interplay between appraisal, coping and emotion, each changing and evolving as the transaction unfolds. Appraisal involves the individual’s evaluations of the situation and his or her own resources, both of which are likely to change during the course of an Antarctic over-winter. Studies of participants in polar crossing teams indicate considerable flexibility in use of coping strategies (Leon et al., 1991, 2011a; Suedfeld et al., 2017). To date, however, few studies have addressed contextual changes in coping strategies among personnel over-wintering on Antarctic stations.

The overall aim of the study was to build on previous work and add to knowledge on processes contributing to psychological resilience in the Antarctic context. Specifically, we monitored coping strategies among personnel spending 10 months on a research station in Antarctica, and examined to what extent these changes coincided with alterations in mood and sleep. We studied the time-based changes in four coping strategies: active problem solving, palliative reactions, avoidance and passive expectancies, and comforting cognitions. Active problem solving is reflective of beliefs about own capacities to handle the situation, a coping strategy that is associated with stress resilience (Levine and Ursin, 1991). In contrast and across a variety of settings, avoidance and passive expectancies have been found to correlate with feelings of distress, depression and physiological stress indicators (Levine and Ursin, 1991; Kashdan et al., 2006). Palinkas and Browner (1995) found an increase in depressive symptoms when they compared scores at baseline with scores after the end of a year in Antarctica, and this change was accompanied by avoidance and emotional discharge as coping strategies. A similar pattern was reported following a space simulation study in which participants lived and worked together in a confined habitat for 105 days (Nicolas et al., 2013). Palliative reactions and comforting cognitions have in common that they serve the purpose of dampening the arousal or stress response although they do not change the stressful situation. As noted by Leon et al. (2011b), the ability to engage in emotionally oriented strategies, such as comforting cognitions, is important in situations where the stressor is chronic or not perceived as controllable as is the case for many of the stressors that expeditioners experience while overwintering in Antarctica. Supporting this view, Grant et al. (2007) found that participants rated by station commanders as “exceptionally well adapted” had higher levels of emotion-focused coping and fewer subjective health complaints than their less well adapted counterparts. Based on the reviewed literature, we expected that: (a) during midwinter participants report poorer sleep quality, less positive affect (PA) and more negative affect; (b) poorer sleep quality, less PA and more negative affect (NA) during midwinter will coincide with a decrease in active problem solving, comforting cognitions and palliative reactions, and increase in avoidance and passive expectancies.

## Materials and Methods

### Participants

The sample consisted of members of two crews overwintering on the Concordia station in Antarctica from the beginning of February until November. Among station members in Crew 1, 14 consented to participate in the study. All of them were males, with ages ranging from 23 to 58 years [*M* = 38.3, *SD* = 10.64]. Eight were Italians and six were French. The study sample from Crew 2 included 13 respondents, among whom three females, the age of the subjects ranged from 22 to 51 years [*M* = 34.5, *SD* = 9.17], seven Italians and six French. Four members of Crew 1 and five members of Crew 2 had previously overwintered on Antarctica. Medical and psychiatric screening was undertaken as part of the selection of participants. The crews also underwent a 2-day long psychological training program prior to departure preparing them for possible psychological and social issues that may arise during the stay. The training program focused on self-reflection, team building processes and coping with communication problems.

### The Physical Environment and the Facilities at the Concordia Station

**FIGURE 1:**
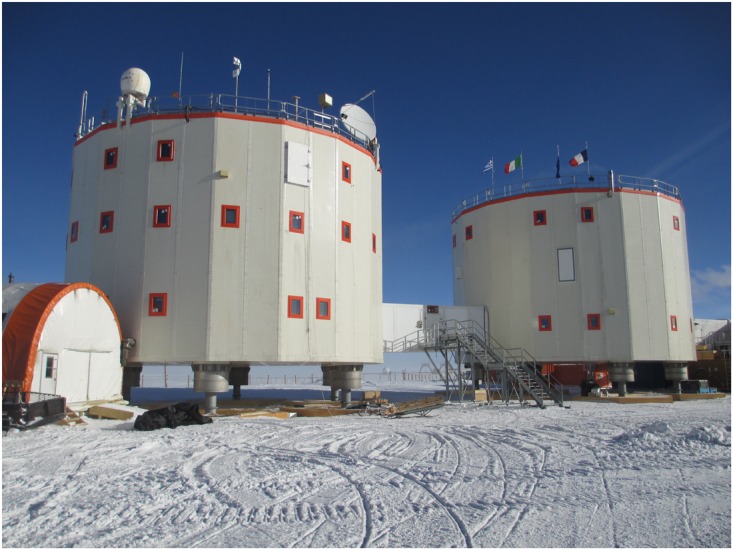
Concordia research base in Antarctica. Photo: ESA.

The Concordia Station (see Figure 1) is a permanently crewed French-Italian research station located at an altitude of 3,232 m at Dome C (75° 06′ S, 123° 23′ E), 1,000 km inland from the coast of the Antarctic Ocean. The physical environment of the station has the driest desert climate on Earth and a low air-pressure and oxygen-poor atmosphere. The mean temperature is -51°C, the lowest recorded temperature is -85°C. Access to the station is only possible from November to February due to the cold and darkness. The station is 1500 m^2^ and comprises three towers connected by enclosed walkways. The first tower, called the quiet building, contains individual sleeping quarters, two bathroom, three toilets, hospital, laboratories (glaciology, meteorology, astronomy). The second tower, dedicated to noisy rooms, houses the canteen, workshop, the waste water treatment plant, social rooms (for video-watching, music), a gym, the kitchen, and storage rooms. The third tower is made up of eleven container size modules where the electric power plant, the boiler room and a second workshop holding devices to measure atmospheric data such as wind speed and humidity, are located. The temperature inside the base is usually between 21 and 23°C.

The base can accommodate about 60 people during the short summer period, and can hold 15-16 crew members during winter. Typically in winter, the crew includes four technicians, a plumber, a cook, a medical doctor and 8-9 scientists typically working in the fields of astronomy and astrophysics, glaciology, atmospheric sciences and geophysics. Time spent outside the base during winter is minimal because of the extreme temperatures which again may lead to under-stimulation and boredom due to the monotony of the indoor facilities. There are few forms of entertainment on the base and daily life could be highly repetitive (Salam, 2009). Communications with the outside world were limited to satellite telephone, restricted email contacts and there was no internet access at the time of the data collection. Temporarily the telecommunication systems could break down and the crew had to be self-reliant without opportunities to seek advice from outside sources.

### Measures

#### Coping Strategies

The UCL (Schreurs et al., 1993) measures different ways of dealing with problems, and consists of 47 items to be answered on a four-point scale from 1 (never or seldom) to 4 (very often). The four scales included in the present research were defined as follows (Gravdal and Sandal, 2006); (1) Active problem solving (7 items): acting immediately and being goal-oriented, sorting things out; (2) Palliative reactions (8 items): engaging in other activities, trying to relax; (3) Avoidance and passive expectancies (8 items): withdrawing from problematic situations; (4) Comforting cognitions (5 items): thinking that worse things happen, or that the situation may not be as bad as it seems. Higher scores on the scales indicate more frequent use of the specific coping strategies. UCL was administered every month during the stay on Concordia.

#### Sleep Diary

The expedition members completed a sleep diary in the first week of each month during the stay. The diary comprises daily estimates of parameters such as bedtime, light out time, sleep onset latency (time between falling asleep and turning off the light), number and duration of awakenings, time of final awakening, rise time, and sleep quality (Carney et al., 2012). In this paper, we only refer to data on the subjective evaluation of sleep quality. Sleep quality was indicated on a five point scale from very bad (1) to very good (5).

#### Affective States

The PANAS (Watson et al., 1988) is a self-report adjective checklist measuring affective states. Two higher-order dimensions of positive affect (PA) and negative affect (NA) are measured by subscales each consisting of 10-items. Items designed to measure PA include feeling active, alert, attentive, determined, enthusiastic, excited, inspired, interested, proud, and strong, while items designed to measure NA include feeling afraid, ashamed, distressed, guilty, hostile, irritated, jittery, nervous, scared, and upset. For each item, subjects rated whether they had felt that way over the past week on a scale from very slightly or not at all (1) to extremely (5). Scores for PA and NA were obtained by averaging the scores on the included items. Completion of the PANAS was scheduled every month during the stay on Concordia.

### Procedure

The Regional Medical Committee for Research Ethics (REK West, Norway) approved the experimental protocol. All subjects were fully informed as to the nature of the investigation and were told their rights, such as the right to withdraw from the experiment at any time, and signed a Declaration of Consent Form providing this information. One of the investigators met with the participants prior to their departure to Antarctica to inform them about the study. A unique code was assigned to each participant in order to monitor longitudinal data and to protect participant anonymity. All questionnaires were administered in the native language of the participants (either French or Italian).

### Data Analysis

Data was analyzed using SPSS statistics 24 (IBM, 2016). We first used repeated measures multivariate analysis of variance to address changes in scores across the 9-month stay at the research situation. The nine measurement occasions were included as repeated measures factor and the scale scores as dependent variables. We then continued to address time course changes using correlational analysis to compare relations between psychological variables in the months prior to (months 1 – 4) and post winter (months 5 – 9). A caveat is warranted before presenting the results of the analyses. Our sample size of 27 participants may be large for a study of consequences of living in such a contrived, harsh environment, but for statistical purposes the sample size is relatively small.

## Results

**FIGURE 2:**
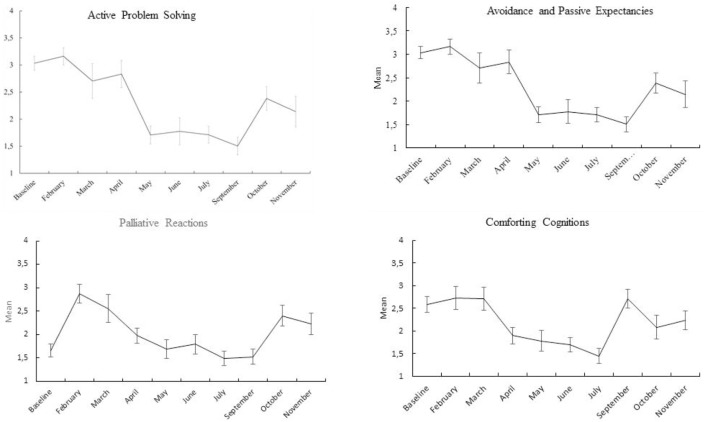
Means and 95% confidence intervals of coping strategies scales at baseline and the 9 months at the research station.

**FIGURE 3:**
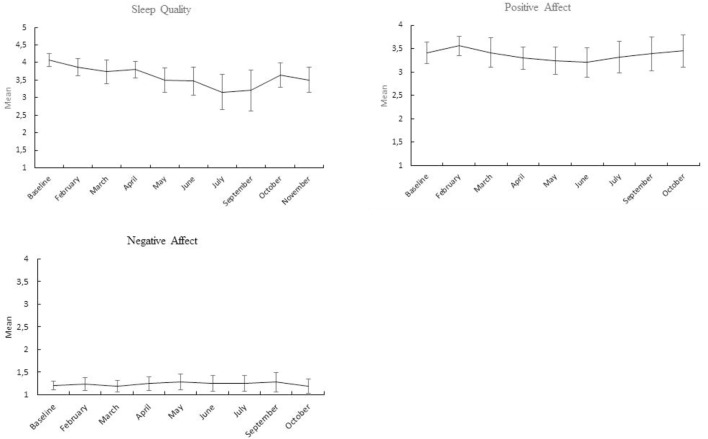
Means and 95% confidence intervals of positive and negative, and subjective sleep quality at baseline and the 9 months at the research station.

Scales means and 95% confidence intervals from the repeated measures multivariate analysis of variance are presented in Figures 2, 3. The first data point in the graphs is the baseline level. As can be seen from Figure 1, NA scores were continuously low during the entire stay at the station and did not change over time. PA and the coping strategies scores are patterned having a minimum around cold and dark mid-winter months. This pattern is indicative of a quadratic relationship between time and the dependent variables. Table 1 presents linear, quadratic and cubic components and their significance. As can be seen, the quadratic equations produced the largest effect sizes, notably for the coping strategies scores and PA, followed by the linear component. Only for subjective sleep quality, the linear equation produced the largest effect size (the quadratic effect just failed to reach significance, *p* = 0.06). Cubic equations tended to produce small effects, only reaching significance for the coping strategy, comforting cognitions.

**Table 1:** Effect sizes of linear and quadratic components in repeated measures analyses of all scales across the 9 months.

Scale	Linear effect	Quadratic effect	Cubic effect
Active problem solving	0.74^∗∗∗^	0.75^∗∗∗^	0.08
Avoidance and passive expectancies	0.57^∗∗∗^	0.82^∗∗∗^	0.06
Palliative reactions	0.57^∗∗∗^	0.77^∗∗∗^	0.00
Comforting cognitions	0.32^∗∗^	0.70^∗∗∗^	0.38^∗∗^
Sleep quality	0.19^∗^	0.13	0.07
Positive affect	0.02	0.43^∗∗∗^	0.13
Negative affect	0.00	0.07	0.08

^∗^*p < 0.05. ^∗∗^*p* < 0.01. ^∗∗∗^*p* < 0.001*.

Findings from the correlational analysis are reported in Table 2. Correlations between the psychological variables and time during the first 4 months were mostly negative (note that NA was reverse scored). In the latter 4 months (i.e., months 5 – 9) correlations were positive. Correlations corroborate findings from the repeated measures analysis and highlight a pattern of depleting resources when going toward the cold period and replenishing of resources in the second half of the overwinter stay.

**Table 2:** Correlations between month number [from February (1) to October (9)] and scale score, separately for the first 4 and last 5 months.

Correlations	First 4 months	Last 5 months
Active problem solving	-0.30	0.04
Avoidance and passive expectancies	-0.36	0.17
Palliative reactions	-0.42	0.13
Comforting cognitions	-0.45	0.39
Sleep quality	-0.19	0.09
Positive affect	-0.22	0.40
Negative affect (reverse scored)	0.01	0.23

## Discussion

The findings from this study suggest that coping strategies, sleep quality, and PA were influenced by the environmental conditions to a smaller or larger degree during midwinter. Quadratic time-based models demonstrated the greatest effect sizes, suggesting that when the conditions are harshest, resources are more depleted and participants were less involved in any form of coping and reported less PA. Also subjective sleep quality showed a negative trend over time, a result consistent with other research (Bhargava et al., 2000; Pattyn et al., 2018) although at the end of the stay the average score did increase slightly. While chronic hypoxia might lead to deterioration in sleep quality in high altitude (Collet et al., 2015), the effect of hypoxia on adaptation among residents on Concordia has been shown to persist over time (Porcelli et al., 2018). Thus, we argue that hypoxia cannot explain seasonal variations in sleep quality observed in this study. The reduction in sleep quality and PA is consistent with the “midwinter syndrome” observed by other researchers (Bhargava et al., 2000; Palinkas and Suedfeld, 2008). It is noticeable that reports of NA remained low over time and did not show the expected change during midwinter. One possible explanation is that participants were reluctant to report distress.

Perhaps the most striking result from this study was the reduction in all of observed coping strategies during the midwinter period. This pattern contradicts the idea that emotional strategies and avoidance take over from more active strategies in situations involving chronic stressors. Our findings may reflect that participants became more indifferent or emotionally flat during the winter months. This interpretation is consistent with early research which noted the occurrence of a mild psychological fugue state known as the Antarctic stare, around the third quarter of the stay (Barabasz et al., 1983). The phenomenon state is characterized by an altered state of consciousness or pronounced absentmindedness, “drifting,” wandering off attention, and deterioration in situational awareness. We believe that this reaction is not unique to people overwintering in Antarctica. For example, during a 520 days confinement study (MARS500) crew members reported reduced need for stimulation around the third quarter (Sandal and Bye, 2015). Interactions between crew members declined, and one crew member showed indications of dissociation. The state of seeking reduced stimulation, and emotional flatness bears resemblance to what could be called “psychological hibernation.” A state of psychological hibernation may be beneficial for coping with the harshness of prolonged exposure to stress in extreme environments. The ability to “switch off” mentally has been associated with positive outcomes in the work stress literature (Sonnentag and Bayer, 2005). However, psychological detachment has also been described as a symptom of burnout after prolonged exposure to stress at work (Maslach and Leiter, 2016). Whilst psychological hibernation could be an adaptive response to the extreme conditions, especially if it disappears when conditions become less extreme (as evidenced by the increase in coping strategy use reported in the present study), understanding the nature of this phenomenon should be an avenue for future research. Further research is also needed to determine the extent to which this state might be associated with decrement in cognitive function and the ability to react to acute, safety-critical situations. So far studies on cognitive performance investigations on Antarctica have been controversial. While no detectable cognitive deterioration was found in a study of a crew overwintering on Concordia (Barkaszi et al., 2016), other researchers have shown that residence in Antarctica had a detrimental effect on cognition (Reed et al., 2001).

We acknowledge that the present findings need to be replicated on a larger or multiple samples. A limitation of this study is that data is based on self-reports (questionnaires), and that responses may be influenced by report biases such as social desirability (although social desirability cannot be used to explain the seasonal patterning of coping). Steinach et al. (2016) found that female over-winterers showed a greater decline in sleep quality than men indicating that they are more susceptible to stressors in the Antarctic. In the present study, gender effects could not be examined due to the small number of female participants (*N* = 3).

Overall, our findings raise further questions regarding the nature of adaptation and the day-to-day and month-to-month functioning of expeditioners in extreme and unusual settings. The existence of “the third quarter phenomenon” characterized by low motivation, interpersonal tension and low mood has been controversial among researchers (Smith and Sandal, 2017). While this study suggests that psychological change indeed occurs in this phase, we question whether this phase is characterized by reduced resilience. Rather we suggest, in line with salutogenic reactions observed in many expedition studies including those in Antarctica (Palinkas and Suedfeld, 2008), that psychological hibernating in itself may represent an adaptive way of coping with the harshness of over-wintering, and therefore associated with psychological resilience in this context. This way of coping is different from the conventional coping classification (problem-focused, emotion-focused, and avoidance) and is mainly characterized by a low level of activation, a decrease in coping and PA, but no change in NA. If hibernation is indeed an adaptive coping mechanism, this may have implications for countermeasures aimed at maintaining psychological resilience in confined environments, such as research stations in Antarctica or long-duration spaceflight. For example, psychological detachment may be facilitated by stress amelioration techniques such as yoga, meditation and self-hypnosis. While we are not aware of empirical studies evaluating the efficiency of such countermeasures in confined environments, anecdotal reports from Antarctic expeditions suggest that people do practice such techniques and that these have been experienced as helpful in maintaining psychological resilience during the harsh winter period (Sharma, 2001; Weiss et al., 2007).

## Author Contributions

GS conceived the study and conducted the data collection. FvdV analyzed the results. NS contributed to theoretical and empirical background. All authors reviewed the manuscript.

## Conflict of Interest Statement

The authors declare that the research was conducted in the absence of any commercial or financial relationships that could be construed as a potential conflict of interest.

## References

[B1] Arendt J. (2012). Biological rhythms during residence in polar regions. *Chronobiol. Int.* 29 379–394. 10.3109/07420528.2012.668997 22497433 PMC3793275

[B2] Barabasz M. Barabasz A. F. Mullin C. (1983). Effects of brief Antarctic isolation on absorption and hypnotic susceptibility: preliminary results and recommendations. *Int. J. Clin. Exp. Hypon.* 31 235–238. 10.1080/00207148308406617 6618726

[B3] Barkaszi I. Takács E. Czigler I. Balázs L. (2016). Extreme environment effects on cognitive functions: a longitudinal study in high altitude in Antarctica. *Front. Hum. Neurosci.* 10:331. 10.3389/fnhum.2016.00331 27445768 PMC4928492

[B4] Bechtel R. B. Berning A. (1991). “The third-quarter phenomenon: do people experience discomfort after stress has passed?,” in *From Antarctica to Outer Space: Life in Isolation and Confinement* Vol. 26 eds Clearwater Y. A. Harrison A. A. McKay C. P. (New York, NY: Springer-Verlag).

[B5] Beute F. de Kort Y. A. (2014). Salutogenic effects of the environment: review of health protective effects of nature and daylight. *Appl. Psychol. Health Well Being* 6 67–95. 10.1111/aphw.12019 24259414

[B6] Bhargava R. Mukerji S. Sachdeva U. (2000). Psychological impact of the Antarctic winter on Indian expeditioners. *Environ. Behav.* 32 111–127. 10.1177/00139160021972450 11542940

[B7] Carney C. E. Buysse D. J. Ancoli-Israel S. Edinger J. D. Krystal A. D. Lichstein K. L. (2012). The consensus sleep diary: standardizing prospective sleep self-monitoring. *Sleep* 35 287–302. 10.5665/sleep.1642 22294820 PMC3250369

[B8] Chen N. Wu Q. Li H. Xu C. (2016). Different adaptations of Chinese winter-over expeditioners during prolonged Antarctic and sub-Antarctic residence. *Int. J. Biometeorol.* 60 737–747. 10.1007/s00484-015-1069-8 26842369

[B9] Collet G. Mairesse O. Cortoos A. Tellez H. F. Neyt X. Peigneux P. (2015). Altitude and seasonality impact on sleep in Antarctica. *Aerosp. Med. Hum. Perform.* 86 392–396. 10.3357/amhp.4159.2015 25945557

[B10] Couch S. R. Coles C. J. (2011). Community stress, psychosocial hazards, and EPA decision-making in communities impacted by chronic technological disasters. *Am. J. Public Health* 101(Suppl. 1), S140–S148. 10.2105/AJPH.2010.300039 21836109 PMC3222505

[B11] Do N. Mino V. L. Merriam G. R. LeMar H. Case H. S. Palinkas L. A. (2004). Elevation in serum thyroglobulin during prolonged Antarctic residence: effect of thyroxine supplement in the polar 3,5,3’-triiodothyronine syndrome. *J. Clin. Endocrinol. Metab.* 89 1529–1533. 10.1210/jc.2003-031747 15070908

[B12] Fletcher D. Sarkar M. (2013). Psychological Resilience. *Eur. Psychol.* 18 12–23. 10.1027/1016-9040/a000124

[B13] Grant I. Eriksen H. R. Marquis P. Orre I. J. Palinkas L. A. Suedfeld P. (2007). Psychological selection of Antarctic personnel: the “SOAP” instrument. *Aviat. Space Environ. Med.* 78 793–800. 17760288

[B14] Gravdal L. Sandal G. M. (2006). The two-factor model of social desirability: relation to coping and defense, and implications for health. *Pers. Individ. Dif.* 40 1051–1061. 10.1016/j.paid.2005.11.004

[B15] IBM (2016). *SPSS Statistics for Windows, Version 24.0*. Armonk, NY: IBM Corp.

[B16] Kashdan T. B. Barrios V. Forsyth J. P. Steger M. F. (2006). Experiential avoidance as a generalized psychological vulnerability: comparisons with coping and emotion regulation strategies. *Behav. Res. Ther.* 44 1301–1320. 10.1016/j.brat.2005.10.003 16321362

[B17] Kjærgaard A. Leon G. R. Venables N. C. Fink B. A. (2013). Personality, personal values and growth in military special unit patrol teams operating in a polar environment. *Mil. Psychol.* 25 13–22. 10.1037/h0094753

[B18] Lazarus R. S. Folkman S. (1984). *Stress, Appraisal and Coping*. New York, NY: Springer.

[B19] LeGates T. A. Fernandez D. C. Hattar S. (2014). Light as a central modulator of circadian rhythms, sleep and affect. *Nat. Rev. Neurosci.* 15 443–454. 10.1038/nrn3743 24917305 PMC4254760

[B20] Leon G. R. Kanfer R. Hoffman R. G. Dupre L. (1991). Interrelationships of personality and coping in a challenging extreme situation. *J. Res. Pers.* 25 357–371. 10.1016/0092-6566(91)90027-N

[B21] Leon G. R. Sandal G. M. Fink B. Ciofani P. (2011a). Positive experiences and personal growth in a two-man North Pole expedition. *Environ. Behav.* 43 710–731. 10.1177/0013916510375039

[B22] Leon G. R. Sandal G. M. Larsen E. (2011b). Human performance in polar environments. *J. Environ. Psychol.* 4 353–360. 10.1016/j.jenvp.2011.08.001

[B23] Levine S. Ursin H. (1991). “What is stress?,” in *Stress- Neurobiology and Neuroendocrinology*, eds Brown M. R. Koob G. F. Rivier C. (New York, NY: Marcel Dekker).

[B24] Lugg D. J. (2005). Behavioral health in Antarctica: implications for long-duration space missions. *Aviat. Space Environ. Med.* 76(6 Suppl.), B74–B77. 15943198

[B25] Maslach C. Leiter M. P. (2016). Understanding the burnout experience: recent research and its implications for psychiatry. *World Psychiatry* 15 103–111. 10.1002/wps.20311 27265691 PMC4911781

[B26] Najjar R. P. Wolf L. Taillard J. Schlangen L. J. M. Salam A. Cajochen C. (2014). Chronic artificial blue-enriched white light is an effective countermeasure to delayed circadian phase and neurobehavioral decrements. *PLoS One* 9:e102827. 10.1371/journal.pone.0102827 25072880 PMC4114570

[B27] Nicholas M. Suedfeld P. Weiss D. S. Gaudino M. (2015). Affective, social, and cognitive outcomes during a 1-Year wintering in Concordia. *Environ. Behav.* 48 1073–1091. 10.1177/0013916515583551

[B28] Nicolas M. Sandal G. M. Weiss K. Yusupova A. (2013). Mars-105 study: time-courses and relationships between coping, defense mechanisms, emotions and depression. *J. Environ. Psychol.* 35 52–58. 10.1016/j.jenvp.2013.05.001

[B29] Palinkas L. A. Browner D. (1995). Effects of prolonged isolation in extreme environments on stress, coping, and depression. *J. Appl. Soc. Psychol.* 25 557–576. 10.1111/j.1559-1816.1995.tb01599.x

[B30] Palinkas L. A. Cravalho M. Browner D. (1995). Seasonal variation of depressive symptoms in Antarctica. *Acta Psychiatr. Scand.* 91 423–429. 10.1111/j.1600-0447.1995.tb09803.x 7676841

[B31] Palinkas L. A. Houseal M. (2000). Stages of change in mood and behavior during a winter in Antarctica. *Environ. Behav.* 32 128–141. 10.1177/00139160021972469 11542941

[B32] Palinkas L. A. Suedfeld P. (2008). Psychological effects of polar expeditions. *Lancet* 371 153–163. 10.1016/s0140-6736(07)61056-3 17655924

[B33] Palmia G. (1963). Psychological observations on an isolated group in Antarctica. *Br. J. Psychiatry* 131 651–654. 10.1192/bjp.109.460.364

[B34] Pattyn N. Van Puyvelde M. Fernandez-Tellez H. Roelands B. Mairesse O. (2018). From the midnight sun to the longest night: sleep in Antarctica. *Sleep Med. Rev.* 37 159–172. 10.1016/j.smrv.2017.03.001 28460798

[B35] Porcelli S. Marzorati M. Healey B. Terraneo L. Vezzoli A. Bella S. D. (2018). Author correction: lack of acclimatization to chronic hypoxia in humans in the Antarctica. *Sci. Rep.* 8:7063. 10.1038/s41598-018-24804-2 29717142 PMC5931617

[B36] Reed H. L. Reedy K. R. Palinkas L. A. Van Do N. Finney N. S. Case H. S. (2001). Impairment in cognitive and exercise performance during prolonged Antarctic residence: effect of thyroxine supplementation in the polar triiodothyronine syndrome. *J. Clin. Endocrinol. Metab.* 86 110–116. 10.1210/jcem.86.1.7092 11231986

[B37] Salam A. (2009). *The Coldest Job on Earth*, Vol. 338 London: BMJ 10.1136/bmj.b2453

[B38] Sandal G. M. Bye H. H. (2015). Value diversity and crew relationships during a simulated space flight to Mars. *Acta Astronaut.* 114 164–173. 10.1016/j.actaastro.2015.05.004

[B39] Sandal G. M. Endresen I. M. Værnes R. Ursin H. (1999). Personality and coping strategies during submarine missions. *Mil. Psychol.* 11 381–404. 10.1207/s15327876mp1104_3 11543156

[B40] Schreurs P. J. G. Van der Willige G. Brosshot J. F. Grau G. (1993). *Utrecht Coping List: Handbook*, 2nd Edn. Lisse: Swets & Zeitlinger.

[B41] Sharma S. S. (2001). *Breaking the Ice in Antarctica: The First Indian Wintering in Antarctica*. New Delhi: New Age International Limited.

[B42] Smith N. Kinnafick F. Saunders B. (2017). Coping strategies used during an extreme Antarctic expedition. *J. Hum. Perform. Extrem. Environ.* 13. 10.7771/2327-2937.1078

[B43] Smith N. Sandal G. M. (2017). Third quarter phenomenon: the psychology of life in space. *Room Space J.* 3 52–57.

[B44] Sonnentag S. Bayer U. V. (2005). Switching off mentally: predictors and consequences of psychological detachment from work during off-job time. *J. Occup. Health Psychol.* 10 393–414. 10.1037/1076-8998.10.4.393 16248688

[B45] Steinach M. Kohlberg E. Maggioni M. A. Mendt S. Opatz O. Stahn A. (2016). Sleep quality changes during overwintering at the German Antarctic stations neumayer II and III: the gender factor. *PLoS One* 11:e0150099. 10.1371/journal.pone.0150099 26918440 PMC4769303

[B46] Stuster J. Bachelard C. Suedfeld P. (2000) The relative importance of behavioral issues during long-duration ICE missions. *Aviat. Space. Environ. Med.* 71 A17–A25. 10993304

[B47] Suedfeld P. Shiozaki L. Archdekin B. Sandhu H. Wood M. (2017). The polar exploration diary of Mark Wood: a thematic content analysis. *Polar J.* 7 227–241. 10.1080/2154896X.2017.1333327

[B48] Watson D. Clark L. A. Tellegen A. (1988). Development and validation of brief measures of positive and negative affect: the PANAS scales. *J. Pers.* 64 737–774. 10.1111/j.1467-6494.1996.tb00943.x 3397865

[B49] Weiss K. Feliot-Rippeault M. Gaud R. (2007). Uses of places and setting preferences in a French Antarctic station environment. *Environ. Behav.* 39 147–164. 10.1177/0013916505285934

